# Comparison of different suture techniques for laparoscopic vaginal cuff closure

**DOI:** 10.1038/s41598-024-55586-5

**Published:** 2024-02-28

**Authors:** Christiane E. Förster, Iliana Calabretti, Laura Gubser, Andreas Schötzau, Bernhard Fellmann-Fischer, Viola Heinzelmann-Schwarz, Tibor A. Zwimpfer

**Affiliations:** 1 Department of Gynecological Oncology, University Hospital Basel, 4056 Basel, Switzerland; 2 Medical Faculty, University of Basel, 4056 Basel, Switzerland; 3 Cancer Research, Peter MacCallum Cancer Centre, Melbourne, VIC 3000 Australia

**Keywords:** Laparoscopy, Education, Suturing techniques, Barbed suture, Extracorporeal and intracorporeal knots, Vaginal cuff closure, Surgery, Therapeutic endoscopy, Health care, Health occupations, Outcomes research

## Abstract

Laparoscopic hysterectomy is a commonly performed procedure. However, one high-risk complication is vaginal cuff dehiscence. Currently, there is no standardization regarding thread material or suturing technique for vaginal cuff closure. Therefore, this study aimed to compare extracorporeal and intracorporeal suturing techniques for vaginal cuff closure using a pelvic trainer model. Eighteen experts in laparoscopic surgery performed vaginal cuff closures with interrupted sutures using intracorporeal knotting, extracorporeal knotting and continuous, unidirectional barbed sutures. While using an artificial tissue suturing pad in a pelvic trainer, experts performed vaginal cuff closure using each technique according to block randomization. Task completion time, tension resistance, and the number of errors were recorded. After completing the exercises, participants answered a questionnaire concerning the suturing techniques and their performance. Experts completed suturing more quickly (*p* < 0.001, *p* < 0.001, respectively) and with improved tension resistance (*p* < 0.001, *p* < 0.001) when using barbed suturing compared to intracorporeal and extracorporeal knotting. Furthermore, the intracorporeal knotting technique was performed faster (*p* = 0.04) and achieved greater tension resistance (*p* = 0.023) compared to extracorporeal knotting. The number of laparoscopic surgeries performed per year was positively correlated with vaginal cuff closure duration (*p* = 0.007). Barbed suturing was a time-saving technique with improved tension resistance for vaginal cuff closure.

## Introduction

Total hysterectomies are one of the most commonly performed gynecological procedures globally^1^, and there has been a tendency towards laparoscopic approaches in benign cases^2,3^. In Switzerland, more than 50% of all hysterectomies of benign origin are performed laparoscopically^4^. The American Congress of Obstetricians and Gynecologists strongly recommends a minimally invasive approach to increase the patient benefits and to reduce hospitalization costs^5^.

The risk of vaginal cuff dehiscence (VCD) is increased in laparoscopic compared to abdominal or vaginal hysterectomies, likely due to electrosurgical colpotomy. Previous studies have calculated the overall risk for VCD as 0.64–4.93% for the laparoscopic approach, 0.12% for the abdominal approach, and 0.29% for the vaginal approach^5–8^. There is institutional and individual preference for how vaginal cuff closure (VCC) is performed, especially in minimally invasive hysterectomies. The laparoscopic intracorporeal VCC has shown a significant reduction in VCD compared to transvaginal VCC after total laparoscopic hysterectomy^9^. Additionally, there are different suture materials and techniques that can affect surgical time and strength of the suture, which, in turn, can lead to dehiscence^10^. Given that the operating room (OR) is a major driver of hospital costs, surgical duration is an expensive resource and responsible for higher costs^5,11,12^. In an era of rising costs in medicine and declining reimbursement, it is essential to optimize the effectiveness of the OR by minimizing the costs of necessary, but unprofitable procedures^13^.

The barbed suture is the most recent innovation in thread material that reduces operative time, blood loss, and VCD in minimal invasive hysterectomies. One barbed suture type is the V-Loc™, which is a monofilament, absorbable thread with small unidirectional circumferential barbs that do not require knotting^14,15^. A meta-analysis by Bogliolo et al.^16^ showed that barbed sutures are safe, as well tolerated as traditional sutures, and associated with reduced durations of laparoscopic vaginal vault closures. A number of studies compared it to other traditional techniques, mostly continuous cuff closures and to closure devices such as the Endo Stich^17–20^. However, to our knowledge, no study has compared the barbed suture to intracorporeal and extracorporeal suturing techniques in relation to knot strength and cuff closure spread ability as well as suturing time.

Therefore, the aim of this study was to compare different types of suture materials and knotting techniques using Vicryl 2/0 (intracorporeal knotting), Vicryl 2/0 (extracorporeal knotting), and V-Loc 180 3/0 in cuff closures using an ex-vivo model and to examine whether the extracorporeal knotting technique had comparable benefits in reducing operating times and tension resistance.

## Methods

### Study design

**Figure 1:**
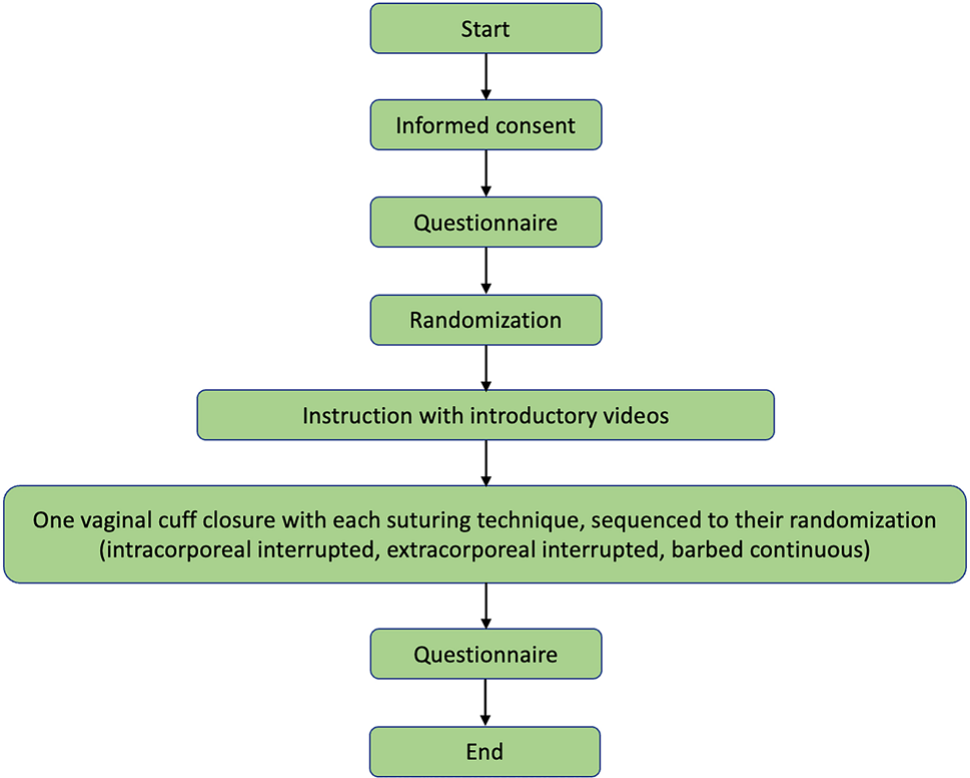
Flow diagram of the study design.

We conducted a randomized study at the University Hospital Basel from the 1^st^ November 2021 to 30th April 2022. The required sample size was estimated using a pragmatic approximation. Eighteen participants were randomized in block randomizations of three. All participants performed interrupted intracorporeal, extracorporeal, and continuous barbed suturing for VCC using each technique according to their randomization. For the primary endpoint, the time required to complete a task was recorded during each participant’s performance. Following the task, the secondary endpoints (i) precision, (ii) knot strength, (iii) cuff closure spread ability, and (iv) number of mistakes made were measured. Before and after completing the tasks, participants were given questionnaires. The first asked about their background and the second about their experience while completing the exercises (Fig. 1). All methods were carried out in accordance with the CONSORT statement guidelines.

### Study population

In total, 18 experts were successfully recruited with a dropout rate of zero, and 54 measurements were obtained. Qualifying as an expert required one to be a surgeon with more than five years of operative experience and more than thirty laparoscopic interventions per year. Study participants were recruited from one tertiary and three secondary hospitals. All participants gave their written, informed consent to participate in the study. The anonymization of personal data was guaranteed.

### Instrument set-up

All exercises were carried out on a box trainer. An endoscopy tower was equipped with a 24-inch monitor and a 300 W Xenon light source (Karl Storz SE & Co., Tuttlingen, Germany) and a Storz Hopkins II, 10 mm, 0° telescope with a Xenon Nova 300 light source and an Image 1 H3-Z Full HD camera (Karl Storz SE & Co., Tuttlingen, Germany) was used. Two access points equivalent to the lateral ancillary trocar entry points were used for the instruments. Two needle holders (Geyl Medical 801.023), laparoscopic scissors, and a closed jaw type knot pusher (Karl Storz 26596 D closed jaw end) were used.

### Exercises

The colpotomy model was made of mesh-augmented silicone with a similar shape and size as a real colpotomy and was set up on the posterior wall of the box trainer. A brief instructional video showed the three different suturing techniques. After a short individual warm-up (20 min maximum) the experts began the different suturing techniques according to their randomization, completing one run per suture type for three runs in total.

#### Vaginal cuff closure with intracorporeal interrupted suturing

Suture A was a closure technique using three interrupted figure-eight sutures and intracorporeal knotting with a polyfilament thread (Vicryl, polyglactin 910, Johnson & Johnson). These three sutures were performed with a surgeon’s knot, which translated to securing the knot with three loops (Video 1).

#### Vaginal cuff closure with extracorporeal interrupted suturing

Suture B used the same closure technique as suture A but with knotted extracorporeal polyfilament thread (Vicryl, polyglactin 910, Johnson & Johnson). The three interrupted figure-eight sutures were performed with a surgeon’s knot. The tightening of the knots was made with one hand and tightened with a knot pusher (Video 2).

#### Vaginal cuff closure with barbed continuous suturing

Suture C was a continuous, unidirectional barbed suture made with V-Loc™ (180 Absorbable Wound Closure Device; Covidien, Mansfield, MA, USA). This suture was made unidirectional. Thus, after the first stitch, the thread was pulled through the loop. After 5 more stitches from right to left, the thread was cut at the end of the colpotomy without extra anchoring stitches (Video 3).

### Questionnaires

Participants answered questionnaires before and after the exercises. The questionnaire given before the exercises concerned general participant characteristics including sex, age, whether and how often they played video games, what types of sports and instruments they played, and their background regarding surgical and technical skills. After completing the exercises, participants answered a questionnaire regarding how they felt, both mentally and physically, about their experience with the different suturing techniques.

### Statistical analyses

Descriptive statistics are presented as counts and frequencies for categorical data. For metric variables, means with standard deviations, medians, and interquartile ranges were used. Linear mixed-effects models were used to predict spreading capacity with technique and run as predictor variables. Results are presented as mean differences. For total run time and changes in knot strength, the variables were log transformed, and the results presented as geometric mean ratios. A p-value < 0.05 was considered significant. The statistical software R (version 4.1.3) was used for the analyses.

### Ethical approval and consent to participate

All study activities were conducted in accordance with Institutional Review Board (IRB) guidelines for exempt studies. All methods were carried out in accordance with relevant guidelines and regulations. A formal IRB certification of exemption (Req-2021-01075) was provided by the ethics committee of Northwest and Central Switzerland (EKNZ) on the 21st of September 2021. The EKNZ can confirm that the research project (Req-2021-01075) fulfilled the general ethical and scientific standards for research with human subjects. All participants gave their written informed consent to participate in this study. The anonymization of personal data was guaranteed.

## Results

### Participant characteristics

In total, 18 experts were included with 66.6% (12/18) being male and 33.3% (6/18) female. The mean age was 40 years. Six experts were trained gynecologists, 11 were trained surgeons, and one expert had completed training in both specialties. On average, the experts were trained in laparoscopic procedures for 10.2 years (range: 5–20) years and performed 83 (30–250) laparoscopies annually (Table 1).

**Table 1:** General expert characteristics and questionnaire results.

Characteristics	All patients (n = 18) n (%)
Age (years)	
Median	33
Range	(32–50)
Gender (m/f)	
Male	12
Female	6
Medical specialty	
Gynaecology	6
Surgery	11
Gynaecology and Surgery	1
Experience (years)	
Median	10.2
Range	(5–20)
Laparoscopic surgeries (per year)	
Median	83
Range	(30–250)
Right/Left-handed	
Right	15 (83.3%)
Left	3 (16.7%)
Preferred suture material/technique	
V-loc	9 (50%)
Vicryl, intracorporeal	2 (11.1%)
Vicryl, extracorporeal	3 (16.7%)
Other	4 (22.2%)
Estimated colpotomy closure time (minutes)	
Median	12.6
Range	(5–30)
Gaming (yes/no)	
Yes	5 (27.8%)
No	13 (72.2%)
Ball sport hobby (yes/no)	
Yes	10 (55.6%)
No	8 (44.4%)
Musical Instrument (yes/no)	
Yes	8 (44.4%)
No	10 (55.6%)

n, number.

### Time

**Figure 2:**
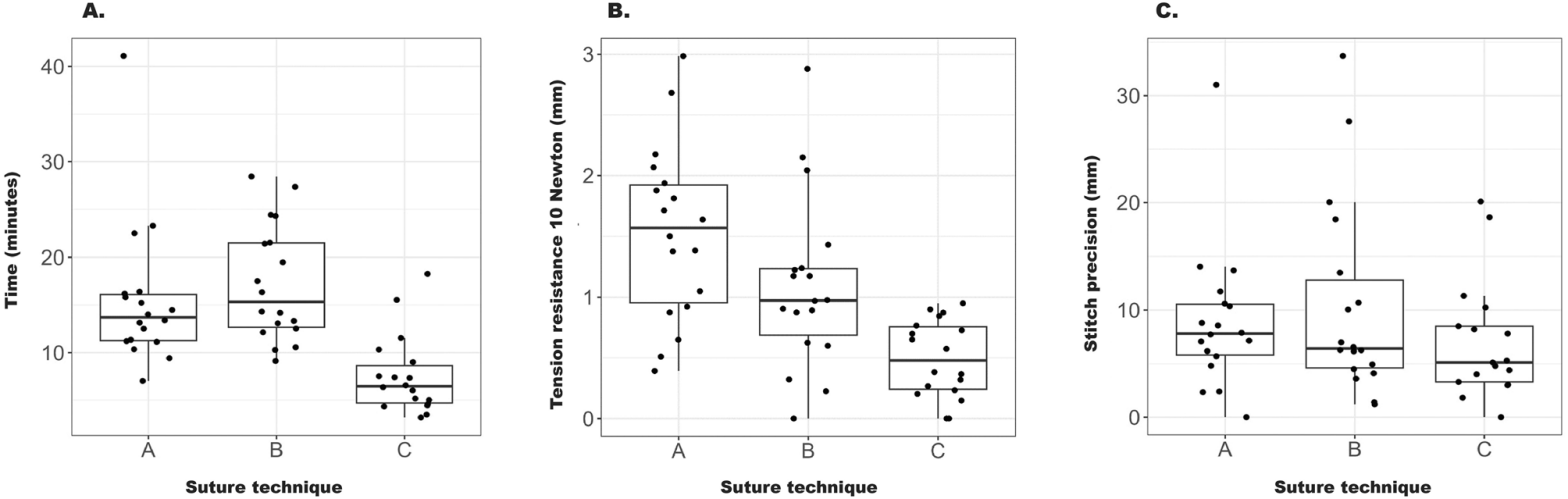
A comparison of the three different suturing techniques for (**A**) time, (**B**) tension resistance, and (**C**) precision according to the geometric mean ratio.

The barbed suture (suture C) led to significantly faster times with a mean of 7.55 min compared to the intracorporeal suture (suture A; mean = 15.46 min; *p* < 0.001) and extracorporeal knotting (suture B; mean = 17.23 min; *p* < 0.001). Furthermore, there was a significant difference between the interrupted A and B sutures (*p* = 0.04). Sutures A and B were consistently slower compared to suture C independent of the first, second, or third run, and the median time for the extracorporeal knot suture varied widely between the experts. However, the experts sutured significantly faster in the third run compared to the first and second run (run 1/2 *p* = 0.899, run 1/3 *p* = 0.048, and run 2/3 *p* = 0.036) (Tables 2 and 3; Fig. 2).

**Table 2:** Comparison of different suturing techniques by time, knot strength, tension resistance, precision, and mistakes.

Parameter	Contrast	*p*-value^a^
Time	Intracorporeal interrupted/Extracorporeal interrupted	0.04
	Intracorporeal interrupted/Barbed continuous	< 0.001
	Extracorporeal interrupted/Barbed continuous	< 0.001
Stitch precision	Intracorporeal interrupted/Extracorporeal interrupted	0.811
	Intracorporeal interrupted/Barbed continuous	0.12
	Extracorporeal interrupted/Barbed continuous	0.132
Tension resistance		
0 Newton	Intracorporeal interrupted/Extracorporeal interrupted	0.832
	Intracorporeal interrupted/Barbed continuous	0.002
	Extracorporeal interrupted/Barbed continuous	0.003
10 Newton	Intracorporeal interrupted/Extracorporeal interrupted	0.099
	Intracorporeal interrupted/Barbed continuous	< 0.001
	Extracorporeal interrupted/Barbed continuous	0.003
Knot strength		
Knot 1		
5 Newton	Intracorporeal interrupted/Extracorporeal interrupted	0.034
	Intracorporeal interrupted/Barbed continuous	0.003
	Extracorporeal interrupted/Barbed continuous	< 0.001
10 Newton	Intracorporeal interrupted/Extracorporeal interrupted	0.032
	Intracorporeal interrupted/Barbed continuous	< 0.001
	Extracorporeal interrupted/Barbed continuous	< 0.001
15 Newton	Intracorporeal interrupted/Extracorporeal interrupted	0.093
	Intracorporeal interrupted/Barbed continuous	< 0.001
	Extracorporeal interrupted/Barbed continuous	< 0.001
Knot 2		
5 Newton	Intracorporeal interrupted/Extracorporeal interrupted	0.53
	Intracorporeal interrupted/Barbed continuous	0.017
	Extracorporeal interrupted/Barbed continuous	0.599
10 Newton	Intracorporeal interrupted/Extracorporeal interrupted	0.083
	Intracorporeal interrupted/Barbed continuous	0.018
	Extracorporeal interrupted/Barbed continuous	0.348
15 Newton	Intracorporeal interrupted/Extracorporeal interrupted	0.135
	Intracorporeal interrupted/Barbed continuous	0.003
	Extracorporeal interrupted/Barbed continuous	0.03
Knot 3		
5 Newton	Intracorporeal interrupted/Extracorporeal interrupted	0.599
	Intracorporeal interrupted/Barbed continuous	0.026
	Extracorporeal interrupted/Barbed continuous	0.058
10 Newton	Intracorporeal interrupted/Extracorporeal interrupted	0.94
	Intracorporeal interrupted/Barbed continuous	0.083
	Extracorporeal interrupted/Barbed continuous	0.045
15 Newton	Intracorporeal interrupted/Extracorporeal interrupted	0.523
	Intracorporeal interrupted/Barbed continuous	0.109
	Extracorporeal interrupted/Barbed continuous	0.006
Mistakes	Intracorporeal interrupted/Extracorporeal interrupted	0.773
	Intracorporeal interrupted/Barbed continuous	1
	Extracorporeal interrupted/Barbed continuous	1

^a^The *p*-values were calculated using a mixed-effects model. A *p*-value < 0.05 was considered significant.

**Table 3:** Overall descriptive statistics of the measured parameters time, stitch precision, and tension resistance (0 and 10 Newton) for all three laparoscopic knot techniques for every run.

Parameter	Technique	Median	Mean	SD^a^	IQR^b^
Total time (min)	Intracorporeal interrupted	13.69	15.46	7.58	4.86
	Extracorporeal interrupted	15.32	17.23	6.08	8.81
	Barbed continuous	6.46	7.55	4.1	3.94
Stitch precision (mm)	Intracorporeal interrupted	7.8	8.89	6.68	4.72
	Extracorporeal interrupted	6.4	10.33	9.07	8.18
	Barbed continuous	5.12	7.03	5.52	5.19
Tension resistance (mm) 0 Newton	Intracorporeal interrupted	1.49	1.83	1.36	1.43
	Extracorporeal interrupted	1.6	1.94	1.73	1.18
	Barbed continuous	0	0.32	0.48	0.84
Tension resistance (mm) 10 Newton	Intracorporeal interrupted	6.25	6.12	2.86	3.87
	Extracorporeal interrupted	3.88	4.15	2.75	2.4
	Barbed continuous	0.86	0.81	0.9	1.16

^a^SD, standard deviation.

^b^IQR, interquartile range.

### Stitch precision

The cumulatively added distance of stitch imprecision for each suture technique was 8.91 mm for suture A, 10.33 mm for suture B, and 7.06 mm for suture C. Concerning the added distance deviation, there was no significant difference between the sutures themselves (A/B *p* = 0.811, A/C *p* = 0.12 and B/C *p* = 0.132) or between the individual runs (*p* > 0.05).

### Tension resistance

The colpotomy cuff without traction had a mean baseline looseness between the figure-eight knots of sutures A and B, which was significantly more than in suture C (A/C *p* = 0.002, B/C *p* = 0.003). There was no significant difference between sutures A and B (*p* = 0.832). The mean cuff gap in suture A was 0.458 mm, 0.487 mm in suture B, and 0.151 mm in suture C. Similarly, under 10 N traction, suture C (0.495 mm) showed significantly less mean cuff dehiscence compared to sutures A (1.53 mm) or B (1.095 mm) (A/B *p* = 0.099, A/C *p* < 0.001, B/C *p* = 0.003).

### Knot strength

The knot strength of the first knot in suture C compared to A and B under a 5 N pull was significantly stronger (A/C *p* = 0.003, B/C *p* < 0.001), while B knot strength was significantly stronger than A (A/B *p* = 0.034). Under 10 N and 15 N conditions, the same results, with C being significantly stronger, was observed (A/C *p* < 0.001, B/C *p* < 0.001). The knot strength of the second knot showed that suture C was significantly stronger than A under 5, 10 and 15 N (*p* = 0.017, *p* = 0.018, and *p* = 0.003, respectively). Suture C was also significantly stronger than suture B under 15 N (*p* = 0.03). The knot strength of the third knot showed a significant difference between C and B under 10 and 15 N (*p* = 0.045, *p* = 0.006), and a significant difference between C and A under 5 N (*p* = 0.026). The p-values of the other comparisons were not significant.

### Mistakes

In total, there were two errors, and both were statistically insignificant (*p* = 0.71).

### Questionnaire results

General demographic parameters such as gender, age and leisure activities (gaming, instruments, or ball sports) did not have significant effects on the simulation outcomes (*p* > 0.05). In addition, the medical specialty (gynecology versus surgery) did not demonstrate a significant impact on tension resistance or time (*p* = 0.413 and *p* = 0.298, respectively). However, experts that were already habituated to the V-Loc™ suture were significantly faster than those not as experienced with the suture material (*p* = 0.003). A positive, significant association between precision and a faster suture time was observed (*p* = 0.004). Experts who are performing more laparoscopic surgeries per year were significantly faster in completing the tasks (*p* = 0.007). Importantly, the surgeon’s years of experience did not correlate with suturing time (*p* = 0.293) (Table 4).

**Table 4:** Correlations between the parameters from the questionnaire and the outcomes of run time, stitch precision, and tension resistance.

Outcome parameter	Self-assessment in the questionnaire	*p*-value^a^
Mean suture time	Habituation to V-loc suture	0.003
	Difficulty with intracorporeal knotting	0.007
	Laparoscopic surgeries per year	0.007
	Difficulty with V-loc handling	0.015
	Time for laparoscopic colpotomy closure	0.241
	Habituation to extracorporeal knotting	0.293
	Experience in years	0.293
	Medical speciality	0.298
	Gender	0.303
	Playing a music instrument	0.362
	Preferred suture technique/material	0.567
	Ball sport hobbies	0.574
	Difficulty with extracorporeal knotting	0.675
	Age	0.681
	Habituation to intracorporeal knotting	0.747
Stitch precision	Time for laparoscopic colpotomy closure	0.004
	Difficulty with intracorporeal knotting	0.068
	Habituation to V-loc suture	0.074
	Medical specialty	0.205
	Ball sport hobbies	0.256
	Laparoscopic surgeries per year	0.436
	Difficulty with V-loc handling	0.529
	Playing a music instrument	0.534
	Preferred suture technique/material	0.534
	Gender	0.640
	Habituation to extracorporeal knotting	0.793
	Experience in years	0.798
	Difficulty with extracorporeal knotting	0.888
	Age	0.955
Tension resistance	Difficulty with intracorporeal knotting	0.056
	Preferred suture technique/material	0.246
	Difficulty with V-loc handling	0.334
	Ball sport hobbies	0.340
	Time for laparoscopic colpotomy closure	0.341
	Medical specialty	0.413
	Playing a music instrument	0.429
	Difficulty with extracorporeal knotting	0.444
	Age	0.473
	Habituation to intracorporeal knotting	0.503
	Habituation to extracorporeal knotting	0.515
	Laparoscopic surgeries per year	0.685
	Habituation to V-loc suture	0.925
	Gender	0.925
	Experience in years	0.974

^a^The *p*-values were calculated using Kruskal–Wallis test or Spearman correlation as appropriate. A *p*-value < 0.05 was considered significant.

## Discussion

The hypothesis that interrupted sutures with extracorporeal knots perform as well as intracorporeal interrupted and continuous barbed sutures for VCCs could not be verified. However, we found that the continuous barbed colpotomy closure technique performed significantly better in terms of time and cuff resistance to tension than interrupted sutures.

Concerning the primary endpoint, the use of the continuous barbed suture resulted in significantly faster closures of the colpotomy cuff. The barbed, continuous suture does not require knots, which likely accounted for the difference in suturing time, yet represents a major technical challenge for many surgeons^21^. In line with our findings, previous research has shown that the use of continuous barbed sutures, compared to interrupted stitches, results in shortened operative time during laparoscopic hysterectomies^10,16,22^. Moreover, it has been demonstrated that the barbed knotless suture is quick and easy to learn, and expert surgeons as well as trainees can perform faster wound closures with this type of thread compared to interrupted sutures^23^, which can lead to saving time in the OR and reducing costs^10,16,22^.

Our data also demonstrated that with each run the experts were faster in finishing their sutures—independent of the suturing technique—with run number three being the overall fastest for all experts. This was also reflected in the questionnaire results where the number of laparoscopic surgeries performed per surgeon was significantly more important in regard to faster suturing times than the overall surgical experience in years.

We found a significantly higher ability to withstand tension for the continuous suture technique compared to the interrupted closure one, independent of intra or extracorporeal knotting. In this instance, the increased tension resistance of the continuous suture technique could potentially translate to a reduced incidence of VCD. VCD is the most severe complication following a hysterectomy and can lead to evisceration. Thus, patients, in most cases, need to be admitted to the hospital for further treatment^5–7^. Therefore, cuff dehiscence is an issue of major concern for gynecologists and different methods of cuff closure have been proposed for improved outcomes. However, to accurately predict the effect of the three different suturing techniques on the incidence of VCD, a prospective randomized trial in vivo needs to be performed.

The literature suggests that interrupted and continuous (barbed or braided) suturing techniques have similar outcomes concerning VCD, irrespective of thread material^16,24,25^. Concerning the thread material, studies have shown that continuous cuff closures had a comparable risk of VCD regardless of the thread material (barbed versus braided/monofilament)^25,26^. Some risk factors associated with VCD are known to be patient-specific such as coitus, cuff infection or hematoma, or chronic coughing in the postoperative period^27,28^ and could not be objectively accounted for in this study. Apart from these parameters, tissue injury related to surgical thermal devices are regarded as other potential causes for VCD. Amongst these devices, it has been shown that ultrasonic dissection causes the least tissue damage, whereas monopolar and bipolar energy may cause deeper tissue necrosis^29^.

The principal limitation of our study was the ex-vivo setting. In comparison, in-vivo tissue is fragile and can bleed, depending on where it is stitched or held by the needle holder, which can influence operation time and the visibility of puncture sites. Furthermore, the colpotomy surrounding ex-vivo tissue is stable and clean without confounding factors such as bleeding, bowel movements, or camera problems. However, all participants in this study had the same conditions, and no confounders influenced the results. Another limitation concerns the comparison between interrupted and continuous suture techniques, which was difficult due to differences in material and physical suture mechanisms; although, the material used in our study is considered to be standard. In addition, in our ex vivo setting, we were not able to measure the risk of the rare but serious complication of bowel obstruction^30,31^ and other long-term outcomes such as vaginal pain and dyspareunia following the use of barbed sutures for VCC^32^.

One strength of our study was the inclusion of 18 experts, which provided an extensive data set of measurements and reduced the selection bias that can occur in trials where only a few surgeons perform operations. Moreover, we were able to compare three different suturing techniques due to the ex-vivo setting. Finally, we were able to measure knot strength and cuff closure spread ability, which would not be possible in a patient setting.

## Conclusions

The results of our study suggest that the barbed suture is a time-saving technique with increased tension resistance for VCC. However, prospective randomized controlled studies in the OR are needed to validate these findings and standardize laparoscopic cuff closures.

### Supplementary Information

Supplementary Information 1.

Supplementary Information 2.

## Data Availability

The datasets analyzed for this study are available at 10.5061/dryad.0gb5mkm5t.

## References

[1] Sutton C (2010). Past, present, and future of hysterectomy. J. Minim. Invasive Gynecol..

[2] Yi Y, Zhang W, Zhou Q, Guo W, Su Y (2011). Laparoscopic-assisted vaginal hysterectomy versus abdominal hysterectomy for benign disease: A meta-analysis of randomized controlled trials. Eur. J. Obstet. Gynecol. Reprod. Biol..

[3] Nieboer TE (2009). Surgical approach to hysterectomy for benign gynaecological disease. Cochrane Database Syst. Rev..

[4] The Federal Office of Public Health. https://www.bag.admin.ch/bag/de/home/zahlen-und-statistiken/zahlen-fakten-zu-spitaelern/qualitaetsindikatoren-der-schweizer-akutspitaeler/qualitaetsindikatoren-fallzahl.exturl.html/aHR0cHM6Ly9zcGl0YWxzdGF0aXN0aWsuYmFnYXBwcy5jaC9wb3/J0YWxfZGUucGhwP3A9cWlmYWxseiZsYW5nPWRlJmJhc2tldD0l/N0NnMy43JTdDMCZxeT0yMDIw.html. Last accessed 2 March 2023.

[5] Committee Opinion No 701: Choosing the Route of Hysterectomy for Benign Disease. *Obstet. Gynecol.* **129**, e155–e159 (2017). 10.1097/AOG.0000000000002112 28538495

[6] Hur HC (2007). Incidence and patient characteristics of vaginal cuff dehiscence after different modes of hysterectomies. J. Minim. Invasive Gynecol..

[7] Nick AM (2011). Rate of vaginal cuff separation following laparoscopic or robotic hysterectomy. Gynecol. Oncol..

[8] Uccella S, Zorzato PC, Kho RM (2021). Incidence and prevention of vaginal cuff dehiscence after laparoscopic and robotic hysterectomy: A systematic review and meta-analysis. J. Minim. Invasive Gynecol..

[9] Uccella S (2018). Laparoscopic versus transvaginal cuff closure after total laparoscopic hysterectomy: A randomized trial by the Italian Society of Gynecologic Endoscopy. Am. J. Obstet. Gynecol..

[10] Smith K, Caceres A (2017). Vaginal cuff closure in minimally invasive hysterectomy: A review of training, techniques, and materials. Cureus.

[11] Shippert RD (2005). A study of time-dependent operating room fees and how to save $100,000 by using time-saving products. Am. J. Cosmet. Surg..

[12] Johns DA (1995). The medical and economic impact of laparoscopically assisted vaginal hysterectomy in a large, metropolitan, not-for-profit hospital. Am. J. Obstet. Gynecol..

[13] Krupka DC, Sandberg WS (2006). Operating room design and its impact on operating room economics. Curr. Opin Anaesthesiol..

[14] Misirlioglu S (2018). Unidirectional barbed suture for vaginal cuff closure without backward stitch in total laparoscopic hysterectomy. J. Obstet. Gynaecol. Res..

[15] Infection Control Today. Covidien Expands V-LOC Family of Absorbable Wound Closure Devices [Internet]. 2010. https://www.infectioncontroltoday.com/view/covidien-expands-v-loc-family-absorbable-wound-closure-devices. Last Accessed 15 January, 2023.

[16] Bogliolo S (2015). Barbed suture in minimally invasive hysterectomy: A systematic review and meta-analysis. Arch. Gynecol. Obstet..

[17] Siedhoff MT, Yunker AC, Steege JF (2011). Decreased incidence of vaginal cuff dehiscence after laparoscopic closure with bidirectional barbed suture. J. Minim. Invasive Gynecol..

[18] Bassi A, Tulandi T (2013). Evaluation of total laparoscopic hysterectomy with and without the use of barbed suture. J. Obstet. Gynaecol. Can..

[19] Morgan-Ortiz F, Contreras-Soto JO, Soto-Pineda JM, Zepeda MA, Peraza-Garay FJ (2013). Comparison between unidirectional barbed and polyglactin 910 suture in vaginal cuff closure in patients undergoing total laparoscopic hysterectomy. Surg. Technol. Int..

[20] López CC (2019). Barbed suture versus conventional suture for vaginal cuff closure in total laparoscopic hysterectomy: Randomized controlled clinical trial. J. Minim. Invasive Gynecol..

[21] Weizman NF, Maurer R, Einarsson JI, Vitonis AF, Cohen SL (2015). Survey on barriers to adoption of laparoscopic surgery. J. Surg. Educ..

[22] Croce E, Olmi S (2000). Intracorporeal knot-tying and suturing techniques in laparoscopic surgery: Technical details. JSLS.

[23] Gözen AS, Arslan M, Schulze M, Rassweiler J (2012). Comparison of laparoscopic closure of the bladder with barbed polyglyconate versus polyglactin suture material in the pig bladder model: An experimental in vitro study. J. Endourol..

[24] Blikkendaal MD (2012). Vaginal cuff dehiscence in laparoscopic hysterectomy: Influence of various suturing methods of the vaginal vault. Gynecol. Surg..

[25] Alessandri F, Remorgida V, Venturini PL, Ferrero S (2010). Unidirectional barbed suture versus continuous suture with intracorporeal knots in laparoscopic myomectomy: A randomized study. J. Minim. Invasive Gynecol..

[26] Tulandi T, Einarsson JI (2014). The use of barbed suture for laparoscopic hysterectomy and myomectomy: A systematic review and meta-analysis. J. Minim. Invasive Gynecol..

[27] Weizman NF, Einarsson JI, Wang KC, Vitonis AF, Cohen SL (2015). Vaginal cuff dehiscence: Risk factors and associated morbidities. JSLS.

[28] Hur HC, Lightfoot M, McMillin MG, Kho KA (2016). Vaginal cuff dehiscence and evisceration: A review of the literature. Curr. Opin Obstet. Gynecol..

[29] Gruber DD, Warner WB, Lombardini ED, Zahn CM, Buller JL (2011). Laparoscopic hysterectomy using various energy sources in swine: A histopathologic assessment. Am. J. Obstet. Gynecol..

[30] Stabile G (2021). Case report: Bowel occlusion following the use of barbed sutures in abdominal surgery. A single-center experience and literature review. Front. Surg..

[31] Limbachiya D, Tiwari R, Kumari R, Aggarwal M (2022). Barbed suture causing acute small bowel obstruction post laparoscopic sacrocolpopexy. CRSLS..

[32] Tsafrir Z (2017). Long-term outcomes for different vaginal cuff closure techniques in robotic-assisted laparoscopic hysterectomy: A randomized controlled trial. Eur. J. Obstet. Gynecol. Reprod. Biol..

