# Supplementary material for: Comparison of different suture techniques for laparoscopic vaginal cuff closure
Source: Sci Rep. 2024 Feb 28;14:4860. doi: 10.1038/s41598-024-55586-5 (PMC10901882; doi:10.1038/s41598-024-55586-5)
Supplement: Supplementary file 1 — Supplementary Information 1. [file 41598_2024_55586_MOESM1_ESM.docx]

**Methods detailed**

There were five outcomes of interest: (a) task time, (b) number of mistakes, (c) precision, (d) knot strength, and (e) cuff closure spread ability.

**Task time**

To measure task completion time, areas were marked to define the start position for the laparoscopic instruments. The task started at this position and was finished once the remains of the thread had been completely removed. A mobile phone with a start/stop feature was used to record the time required for each task to be completed.

**Number of mistakes**

Mistakes were manually counted and recorded.

**Precision**

Precision was measured by the distance, in millimeters, of the suture puncture to the marked puncture using a digital caliper. These 12 distances were then cumulatively added per suture technique and the mean was calculated.

**Cuff closure spreadability**

The spreadability of the cuff closure was measured with a spring balance by attaching both sides and pulling them apart using a force of 10 N. The distance between the colpotomy borders was measured with a digital caliper. This distance was then compared to the colpotomy borders without any pulling force. In this way, the closure could be checked for quality and would uncover fully or insufficiently tightened knots.

**Knot strength**

Suture knot strength was also measured using a spring balance. For intracorporeal and extracorporeal interrupted suturing, if the knot was not tightened correctly with three loops, the knot could freely move, thereby increasing the thread distance. Therefore, one of the thread ends was fixed in a self-made device and the knot was held by a perforated plastic disk while the other end was pulled through the hole. The thread was measured after having been pulled with 0 N, 5 N, 10 N, and 15 N and the added length gain was calculated compared to the baseline (0 N). For barbed continuous suturing with the V-Loc™ loop, the measurement had to be adapted, as it is a thread without a knot. The V-Loc™ thread was marked with a pen longitudinally in the middle along the cuff. The same weights (5 N, 10 N, and 15 N) then pulled on the first, second and third stitch. After pulling, the length increase between the pen mark and the puncture site was measured and noted.
