# Supplementary material for: Comparison of different suture techniques for laparoscopic vaginal cuff closure
Source: Sci Rep. 2024 Feb 28;14:4860. doi: 10.1038/s41598-024-55586-5 (PMC10901882; doi:10.1038/s41598-024-55586-5)
Supplement: Supplementary file 2 — Supplementary Information 2. [file 41598_2024_55586_MOESM2_ESM.docx]

**Videos**

**Video 1** Introductory video of vaginal cuff closure with intracorporeal interrupted sutures. [*https://vimeo.com/813978855*](https://vimeo.com/813978855)

**Video 2** Introductory video of vaginal cuff closure with extracorporeal interrupted sutures. <https://vimeo.com/806128154>

**Video 3** Introductory video of vaginal cuff closure with barbed continuous sutures. <https://vimeo.com/806130274>
